# Serine/threonine protein kinase mediates rifampicin resistance in *Brucella melitensis* through interacting with ribosomal protein RpsD and affecting antioxidant capacity

**DOI:** 10.1128/msystems.01109-24

**Published:** 2024-12-05

**Authors:** Yaqin Yuan, Wenqing Ning, Junjie Chen, Jiquan Li, Tianqi Xue, Cuihong An, Lingling Mao, Guangzhi Zhang, Shizhong Zhou, Jiabo Ding, Xiaowen Yang, Jianqiang Ye

**Affiliations:** 1 Jiangsu Key Laboratory of Zoonosis, Key Laboratory of Jiangsu Preventive Veterinary Medicine, Key Laboratory for Avian Preventive Medicine of Ministry of Education, Jiangsu Co-innovation Center for Prevention and Control of Important Animal Infectious Diseases and Zoonoses, College of Veterinary Medicine, Yangzhou University, Yangzhou, China; 2 Key Laboratory of Animal Biosafe Risk Prevention and Control (North), Ministry of Agriculture and Rural Affairs, Institute of Animal Science, Chinese Academy of Agricultural Sciences, Beijing, China; 3 Department of Preventive Veterinary Medicine, College of Veterinary Medicine, Shandong Agricultural University, Taian, Shandong, China; 4 Tongliao Mongolian Medical Hospital (Tongliao Mongolian Medical Research Institute), China Center for Disease Control and Prevention, Institute of Infectious Disease Control and Prevention, Co-construction research base for brucellosis, Tongliao City, China; 5 Qinghai Institute for Endemic Disease Prevention and Control, Xining, China; 6 Department of Plague and Brucellosis, Shaanxi Center for Disease Control and Prevention, Xi’an, China; 7 Liaoning Province Center for Disease Control and Prevention, Shenyang, China; Iowa State University, Ames, Iowa, USA

**Keywords:** *Brucella*, rifampicin resistance, STPK, *rpsD*, sulfur metabolism

## Abstract

Brucellosis, a zoonotic disease, has re-emerged in both humans and animals, causing significant economic losses globally. Recently, an increasing number of rifampicin-resistant *Brucella* strains have been isolated worldwide without detectable mutations in known antibiotic resistance genes. Here, this study identified the deletion of serine/threonine protein kinase (STPK) gene in *B. melitensis* as an efficient trigger for rifampicin resistance using bioinformatics predictions, a transposon mutant library, and gene mutation strains. Notably, the absence of the STPK could increase the expression of ribosomal proteins and genes involved in sulfur metabolism and reduced glutathione, and decrease NADPH oxidase activity and NADP^+^/NADPH ratio, which is associated with the antioxidant capacity of *B. melitensis*. Moreover, co-immunoprecipitation revealed that STPK could efficiently interact with the ribosomal protein RpsD, possibly altering protein translation and riboswitch expression. These findings demonstrate that the STPK gene mediates resistance by regulating sulfur metabolism to counteract the reactive oxygen species induced by rifampicin. Furthermore, the approaches developed in this study provide a platform for screening new resistance genes in *Brucella* spp., and the identified STPK or its pathway can serve as a potential target for new drug development against rifampicin-resistant *Brucella* spp.

**IMPORTANCE:**

New rifampicin resistance gene in *Brucella melitensis* is identified via bioinformatics predictions and a whole-genome transposon mutant library, new mechanisms of rifampicin resistance in *B. melitensis*, and new function of serine/threonine protein kinase gene and its interaction proteins.

## INTRODUCTION

*Brucella* spp. is a gram-negative facultative intracellular pathogen that causes brucellosis, a zoonotic disease affecting both humans and animals worldwide (1). In humans, brucellosis manifests as fever, fatigue, and joint pain, and it can lead to various complications if not treated promptly (2, 3). In animals, brucellosis can cause orchitis in males and abortion in pregnant females (4). Annually, brucellosis leads to significant health complications in patients and reproductive issues in livestock, causing billions of dollars in economic damages. Currently, brucellosis is re-emerging globally (5, 6), with over 500,000 new cases reported each year (7). Brucellosis remains a significant public health concern in China, with approximately 400 million people at risk of infection. In recent years, there have been more than 70,000 cases of human brucellosis in China (https://www.ndcpa.gov.cn/jbkzzx/c100016/common/list.html). Increased global trade, especially in livestock and animal products, along with international travel, has contributed to the spread of brucellosis (6, 8). The primary *Brucella* species infecting humans and animals include *B. melitensis*, *B. abortus*, *B. suis*, and *B. canis*. Previously, *B. melitensis* biovar 1 was the dominant strain in China, but the main isolate has now change to *B. melitensis* biovar 3 (9, 10). However, new species have been emerging (11), posing a threat to the prevention and control of brucellosis.

Antibiotics remain the most effective treatment for brucellosis. The World Health Organization recommends doxycycline, streptomycin, and rifampicin, which have been utilized clinically since 1986 (12). These are also the first-line drugs for treating brucellosis in China. However, the prolonged use of these antibiotics and the limited variety of drugs increase the risk of developing resistant strains. Studies have shown the emergence of rifampicin-resistant strains in China (13), Qatar (14), and other regions, raising concerns among researchers.

Rifampicin, a rifamycin antibiotic, targets bacterial transcription (15) by binding to the RNA polymerase β-subunit encoded by the *rpoB* gene. Mutations in the *rpoB* gene are a known mechanism of rifampicin resistance in many intracellular pathogens, including *Brucella* spp. (16). However, some rifampicin-resistant strains lack mutations in the *rpoB* gene, suggesting the presence of alternative resistance mechanisms. Proteomic analysis comparing rifampicin-resistant and -sensitive *Brucella* strains identified 39 differentially expressed proteins involved in various metabolic pathways, aside from mutations of *rpoB* gene (17). Additionally, the (p)ppGpp synthetase *Rsh* has been shown to promote rifampicin tolerance in *B. abortus* by regulating the type II toxin-antitoxin module mbcTA (18, 19). Our previous studies have highlighted the role of membrane proteins, particularly type IV secretion system proteins, in rifampicin tolerance in *B. melitensis* (20). Recently, our lab analyzed the rifampicin-intermediate-resistant *Brucella* isolates and found that none of the reported rifampicin resistance-related genes were mutated (21). Thus, this study aims to identify new genes associated with rifampicin resistance in *B. melitensis* through bioinformatics predictions and a transposon mutant library. This study also seeks to elucidate the mechanisms of rifampicin resistance mediated by these new genes. The approaches developed in this study can provide a platform for screening new resistance genes in *Brucella* spp., and the identified genes or pathways can also be used as the potential targets for new drug development against rifampicin-resistant *Brucella* spp.

## MATERIALS AND METHODS

### Acquisition of relevant sequences and culture of *Brucella* strains

In this study, the reference strain used was *B. melitensis* bv.3 Ether, and the wild-type strain (WT) was *B. melitensis* M5. Gene mutation strains were constructed from the WT strain in a BSL-2 laboratory. The genome, amino acid, and nucleic acid sequences of *B. melitensis* were downloaded from the NCBI RefSeq database (ftp://ftp.ncbi.nlm.nih.gov/genomes/). Cultured and inactivated samples of the established transposon mutant library of *B. melitensis* NI were constructed in a BSL-3 laboratory of the National Institute for Communicable Disease Control and Prevention of the Chinese Center for Disease Control and Prevention.

#### Growth curve detection

Growth curve was determined according to a previous study (20). The cultures were incubated at 37°C with shaking at 220 rpm, and the OD_600_ value was determined every 12 h.

### Neural network prediction of rifampicin resistance genes

Following the methodology in reference 22, this study computed various molecular fingerprints of rifampicin. Using the 2D Tanimoto coefficient, similar compounds and their target information were matched from the ChEMBL database (https://www.ebi.ac.uk/chembl/). Deep learning algorithms and neural networks were employed to predict interactions between molecules and targets. The identified protein types were cross-referenced with proteins in the *B. melitensis* genome to select candidate target proteins. The algorithm and model were provided by APExBIO (Houston, TX, USA).

### Screening of new rifampicin resistance genes

Ten microliters of the whole-genome transposon mutant library (23) were cultured in TSB (BD, USA) medium supplemented with rifampicin (4 µg/mL) and kanamycin (50 µg/mL) in 96-well plates at 35°C ± 2°C for 48–96 h. The OD_600_ values were then measured using a microplate reader (Tecan, Switzerland) to select mutants with OD_600_ values above 0.4.

### Molecular docking

The predicted protein structures were downloaded from the AlphaFold Protein Structure Database (https://alphafold.com), and the structure of rifampicin (CID 135398735) was obtained from the NCBI PubChem database (https://pubchem.ncbi.nlm.nih.gov). The protein structures were prepared using MOE (24) (2019) with default parameters, and molecular docking was performed on all sites using default parameters. The molecular docking model with the highest score is used for subsequent analysis.

### Construction of gene deletion and complementation strains

Gene deletion and complementation strains were constructed and identified according to a previous study (20), with some modifications. The manufacturer’s instructions for the Wizard Genomic DNA Purification Kit (Promega, USA) were followed for the extraction of genomic DNA. Sequences of primers for gene deletion and complementation strains are listed in Table S1. PCR products were cloned by ClonExpress MultiS One Step Cloning Kit (Vazyme, China) and transformed into DH5α competent cells (CWbio, China). The positive plasmids were electroporated into *Brucella* strains. In addition to the correct products of PCR amplification, positive colonies should also have the following characteristics. For the deletion strain, it should not grow in the medium containing ampicillin (100 µg/mL); for the complementation strain, it should grow in the medium containing chloramphenicol (25 µg/mL).

### MIC value, MBC value, and *RpoB* gene detection

The detection of the MIC value for rifampicin was performed according to a previous study (20) with some modifications. Briefly, rifampicin was diluted in a 96-well plate with concentrations ranging from 0.25 to 256 µg/mL. Bacterial suspension of WT and gene deletion strains was adjusted to 0.5 McFarland standard (McF) using a turbidimeter (Biomerieux, France), and the gene complementation strain was adjusted to 0.8 McF.

The detection of the minimum bactericidal concentration (MBC) value for rifampicin was performed after MIC value detection according to the methodology in reference (25). A 100 µL cultured medium of the 1/2–16× MIC was selected to TSA medium and incubated at 37°C for 7 days. The medium without any colony growth is the MBC value.

PCR assay of the *rpoB* gene was also performed according to a previous study (20) with some modifications. Twenty microliters of different rifampicin concentrations with *Brucella* strains were cultured on TSA (BD, USA) media or TSA with chloramphenicol (25 µg/mL) and incubated at 35 ± 2°C for 72–96 h, after which 10 colonies were examined.

### Transcriptome and quantitative real-time PCR analysis

Before RNA extraction, twofold volumes of RNAprotect Bacteria Reagent (Qiagen, GER) were added directly to onefold volume of the culture sample. RNA was extracted using TRIzol (Invitrogen, USA) according to the manufacturer’s instructions and treated with DNase I (TaKaRa Bio, Japan) before reverse transcription. The samples were sent to Majorbio Bio-Pharm Technology Co. (China) for sequencing and transcriptome analysis. An adjusted *P* value ≤0.05 and absolute value of log_2_ ratio ≥0.5 were used to identify differentially expressed genes.

cDNAs were synthesized using the PrimeScript RT Reagent Kit (TaKaRa Bio, Japan) according to the manufacturer’s instructions. Quantitative real-time PCR (qPCR) was performed with the primers shown in Table S1 to evaluate the cycle threshold (Ct) value. The value obtained by the relative quantitative method (2^−ΔΔCt^) was used to compare the expression levels of the target genes. Three replicate wells for each gene were evaluated.

### Biochemical parameters assay

Cultures with an OD_600_ of 0.4–0.6 were centrifuged to collect the cells, which were then washed three times with PBS. The cells were resuspended in PBS supplemented with rifampicin (0, 2, 4, and 8 µg/mL) and incubated for 2 h before cell collection for subsequent experiments. Each assay was repeated three times independently.

#### Total ROS measurement

The fluorescent probe 2′,7′-dichlorodihydrofluorescein diacetate (10 µM) was used to assess the production of ROS (26). A Reactive Oxygen Species Assay Kit (Beyotime, China) was used according to the manufacturer’s instructions. After treatment with different concentrations of rifampicin and incubation in the dark for 1 h, fluorescence was measured at an excitation wavelength of 488 nm and an emission wavelength of 525 nm by a microplate reader.

#### NAD^+^/NADH determination

Total NAD^+^ and NADH levels were measured following the manufacturer’s instructions for an NAD^+^/NADH Assay Kit with WST-8 (Beyotime, China). The absorbance was detected at 450 nm. The NAD^+^/NADH ratio was calculated using a standard curve established with the standard products to determine the intracellular NAD^+^ and NADH levels.

#### NADP^+^/NADPH determination

These experiments were conducted on the same samples to measure the levels of total NADP^+^ and NADPH as above described, and their ratios were calculated using an NADP^+^/NADPH Assay Kit with WST-8 (Beyotime, China). The absorbance at 450 nm was measured.

#### GSSG and GSH assay

Following the manufacturer’s protocol for the GSH and GSSG Assay Kit (Beyotime, China), intracellular reduced glutathione (GSH) and oxidized glutathione disulfide (GSSG) levels were measured. A standard curve was established using the standards in the kit. After reacting at room temperature for 25 min, absorbance at 412 nm was measured using a microplate reader. Total glutathione and GSSG levels were calculated using the standard curve, and intracellular GSH levels were determined using the following formula: GSH = total glutathione − GSSG × 2.

#### LDH release assay

The supernatants were collected, and the LDH Cytotoxicity Assay Kit (Beyotime, China) was followed. The samples were incubated at room temperature in the dark for 30–60 min before the absorbance was measured at 490 nm. Maximum release wells and blank control wells were used to calculate the cell death rates.

### H_2_O_2_ assay

The cultures of *Brucella* strains were adjusted to 0.5 McF. One hundred microliters of the adjusted strains was centrifuged to collect the cells, which were then resuspended in TSB medium containing 0, 5, and 10 mM H_2_O_2_. After 1 h incubation at 37°C, viable pathogens were counted, and the survival rate of the strains was calculated.

### *In vitro* eukaryotic protein expression

Primers (Table S1) were designed to clone gene sequences into the pCMV-1 (Myc-tag) and pCMV-7 (3×Flag tag) plasmids. 293T cells in the logarithmic growth were evenly spread into 6-well plates for protein expression. Correctly sequenced plasmids were transfected into the 293T cells using jetPRIME transfection reagent (Polyplus, France) following the manufacturer’s instructions. The cells were cultured for 24–48 h before sample collection. The cells were centrifuged at 1,000 rpm for 5 min, lysed with precooled lysis buffer containing inhibitors, and gently vortexed to ensure thorough lysis. Protein expression was detected via Western blot (WB) analysis with corresponding antibodies (Beyotime, China).

### Pull-down and LC-MS/MS analysis

A Myc-tag Protein IP Assay Kit with Magnetic Beads (Beyotime, China) was used for pull-down assay in this study following the manufacturer’s instructions. Desalted purified proteins were bound to magnetic beads. *Brucella* cultures were collected by centrifugation, quick frozen in liquid nitrogen for 15 min, and lysed twice. Lysates were incubated on ice for 4 h, added to columns with bound protein, and incubated with shaking at 4°C for 4 h before washing several times and eluting interacting proteins. Eluates from columns with only carrier plasmid protein and *Brucella* proteins served as experimental controls.

Nano-LC-MS/MS analysis was performed using an Orbitrap Fusion Tribrid MS (Thermo, USA) instrument equipped with a nanospray flex ion source coupled with a Dionex UltiMate 3000 RSLC nanosystem (Thermo, USA). The raw data files were searched against the *B. melitensis* proteome from the UniProt database using MaxQuant. The proteins and peptides were filtered with a false discovery rate <1%. The enzyme parameters were limited to semitryptic peptides with a maximum miscleavage of 2. Carbamidomethyl (C) of the peptides was set as a fixed modification; oxidation (M) and deamination (NQ) of the N-terminus of the proteins were set as variable modifications.

### Co-IP assay

Co-IP assays were performed using a Flag-tag Protein IP Assay Kit with Magnetic Beads (Beyotime, China). Approximately 2.5 µg of each plasmid was transfected into 293T cells cultured in 1% double antibiotic medium, and the cells were collected after 24–48 h of culture. The collected cells were lysed on ice. A portion of the supernatant was kept as input samples after denaturation, and the remainder was incubated with the prepared magnetic bead suspension overnight at 4°C. After incubation, the beads were washed, and the eluates were denatured as IP samples. WB was performed using the corresponding primary and secondary antibodies, with GAPDH serving as the internal control protein.

### Confocal laser scanning microscopy analysis

HeLa cells at optimal confluency were seeded onto 8-well chamber slides (Beyotime, China), and the plasmids were transfected. The cells were cultured for another 24 h before the old medium was discarded. The cells were gently rinsed with PBS, fixed with 4% PFA, permeabilized with 0.1% Triton X-100 in 5% BSA, and blocked with 5% BSA. Primary and secondary antibodies diluted in PBS were added and incubated at room temperature. The nuclei were stained with DAPI, and the cells were incubated with the antifade mounting medium with DAPI (Beyotime, China). The samples were observed under a confocal laser scanning microscope (Leica, DEU).

### Data analysis and visualization

Statistical analyses were performed using Excel 2010 and SPSS 23.0. A *P* value <0.05 was considered significant when using one-way analysis of variance. Other figures were generated using GraphPad Prism 9. The transcriptome sequencing data were analyzed on the platform of Majorbio Bio-Pharm Technology Co. Ltd. (Majorbio, China).

## RESULTS

### STPK gene deletion increases rifampicin resistance in *B. melitensis*

**Fig 1:**
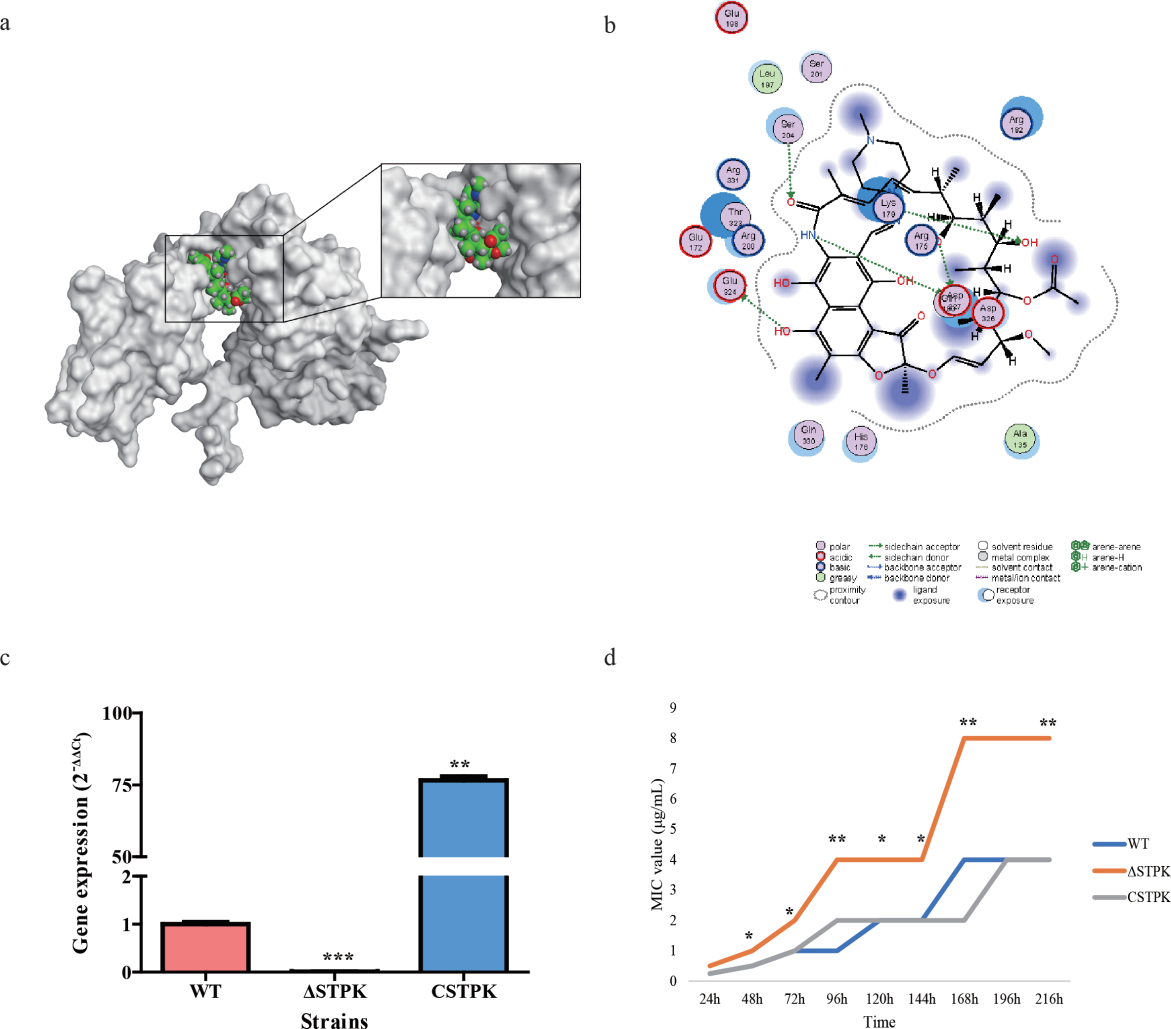
STPK gene and rifampicin resistance in *B. melitensis*. (a) The molecular docking results for STPK protein and rifampicin. This model represents the highest scoring output model. (b) The 2D interaction diagram of STPK protein with rifampicin, showing multiple amino acid sites forming covalent bonds with rifampicin. (c) The results of relative expression levels of STPK in different strains. The WT strain is used as a reference. (d) The MIC value of rifampicin for different strains measured *in vitro*. Significance levels: *P < 0.05, **P < 0.01, ***P < 0.001.

Through bioinformatics predictions and a transposon mutant library, this study identified several novel genes associated with rifampicin resistance. Using neural network predictions to analyze the whole genes of *B. melitensis*, this study obtained 20 target genes and their functions (Table S2). The transposon mutant library, with 6,973 mutants had clear transposon insertions, was also used to screen for new genes associated with rifampicin resistance (Fig. S1a). Among these, nine mutants exhibited an OD_600_ value greater than 0.4 when grown in medium containing 4 µg/mL rifampicin (CLSI-recommended breakpoint for rifampicin resistance in slow-growing strains). Notably, three of these strains corresponded to genes listed in the predictive gene list, with STPK being one of them. Molecular docking revealed that STPK protein can bind to rifampicin (Fig. 1a). Specifically, amino acids Asp327, Glu324, Lys179, and Ser204 form interactions with rifampicin (Fig. 1b). Homologous recombination was used to delete the *STPK* gene, and a gene complementation strain was constructed (Fig. S1b). Gene expression levels of STPK in the WT, deletion (ΔSTPK), and complementation (CSTPK) strains were analyzed (Fig. 1c), confirming successful gene deletion and complementation. The MIC and MBC values for the WT, ΔSTPK, and CSTPK strains were determined *in vitro*. The MIC value of ΔSTPK strain showed rifampicin resistance (4 µg/mL, 96 h), whereas WT (1 µg/mL, 96 h) and CSTPK (2 µg/mL, 96 h) strains remained sensitive to rifampicin (Fig. 1d). The MBC value of the STPK deletion and wild-type strains showed a more significant difference. The MBC value of ΔSTPK strain was 16 µg/mL, while the WT strain was 2 µg/mL, and the CSTPK strain was 4 µg/mL. Sequencing of the *rpoB* gene high-variation region in the deletion strain confirmed that no mutations occurred in the presence of rifampicin at 96 h. All these findings indicate that the deletion of the novel gene STPK increases rifampicin resistance in *B. melitensis*.

### STPK gene deletion enhances GSH production in *B. melitensis* to counteract rifampicin-induced ROS

**Fig 2:**
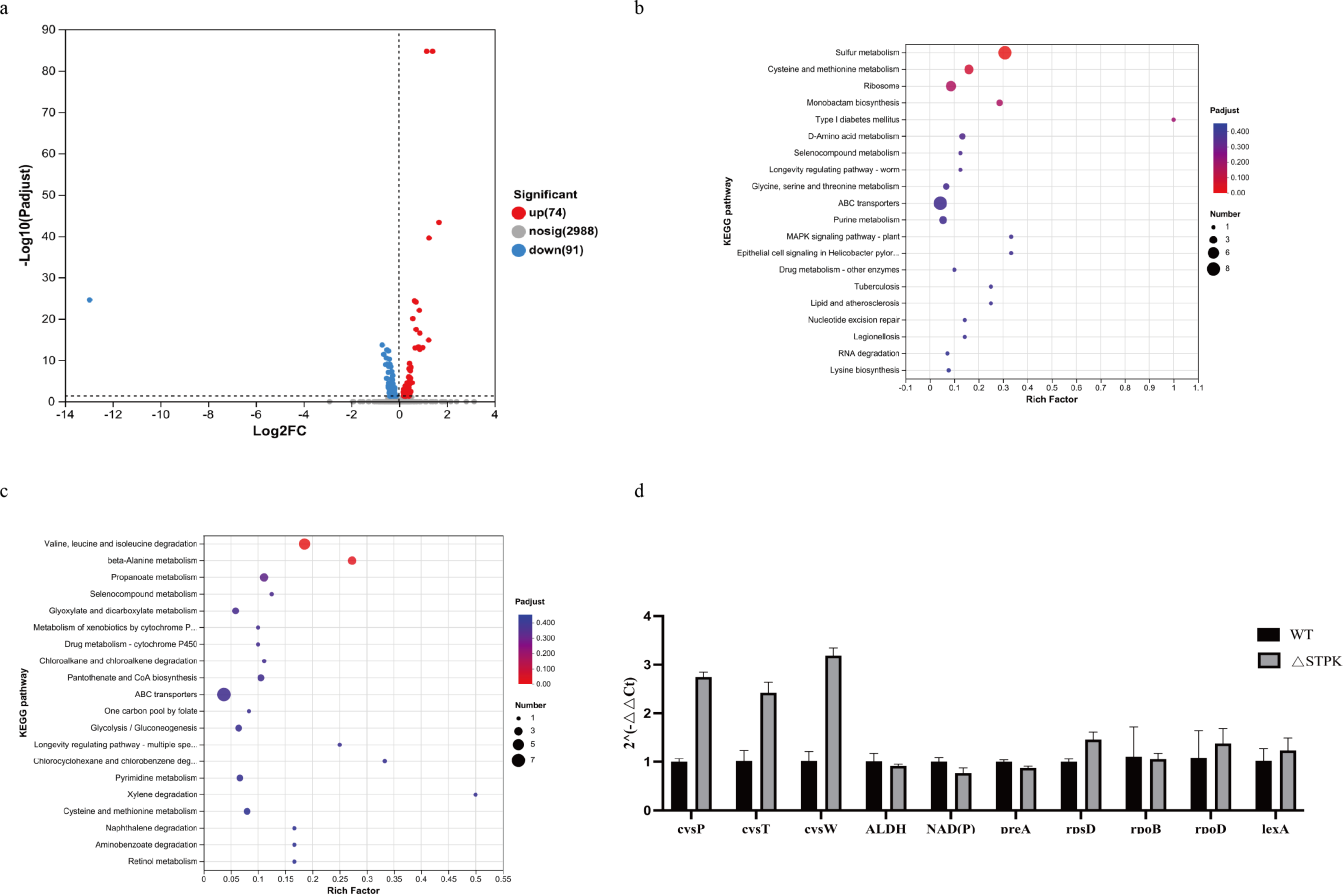
Metabolic impact of STPK deletion in *B. melitensis* as revealed by transcriptome analysis. (a) Volcano plot showing differentially expressed genes in ΔSTPK strain. (b) The KEGG functional cluster of significantly upregulated genes. (c) The KEGG functional cluster of significantly downregulated genes. (d) qPCR validation of transcriptome results, with 16S rRNA as the reference gene.

To investigate the pathways involved in STPK, this study compared the gene expression levels of WT and ΔSTPK strains through transcriptome sequencing. Transcriptome analysis (Table S3) revealed significant changes in gene expression between the ΔSTPK and WT strains. Specifically, 74 genes were upregulated, and 91 genes were downregulated in the STPK deletion strain (Fig. 2a). Notably, genes involved in sulfur metabolism, cysteine and methionine metabolism, and ribosome function were significantly upregulated (Fig. 2b). Conversely, genes involved in valine, leucine, and isoleucine degradation, beta-alanine metabolism, and propanoate metabolism were significantly downregulated (Fig. 2c). The expression levels of some genes were detected by qPCR, confirming the transcriptome results (Fig. 2d). All these demonstrate that STPK affects the metabolism of *B. melitensis*.

**Fig 3:**
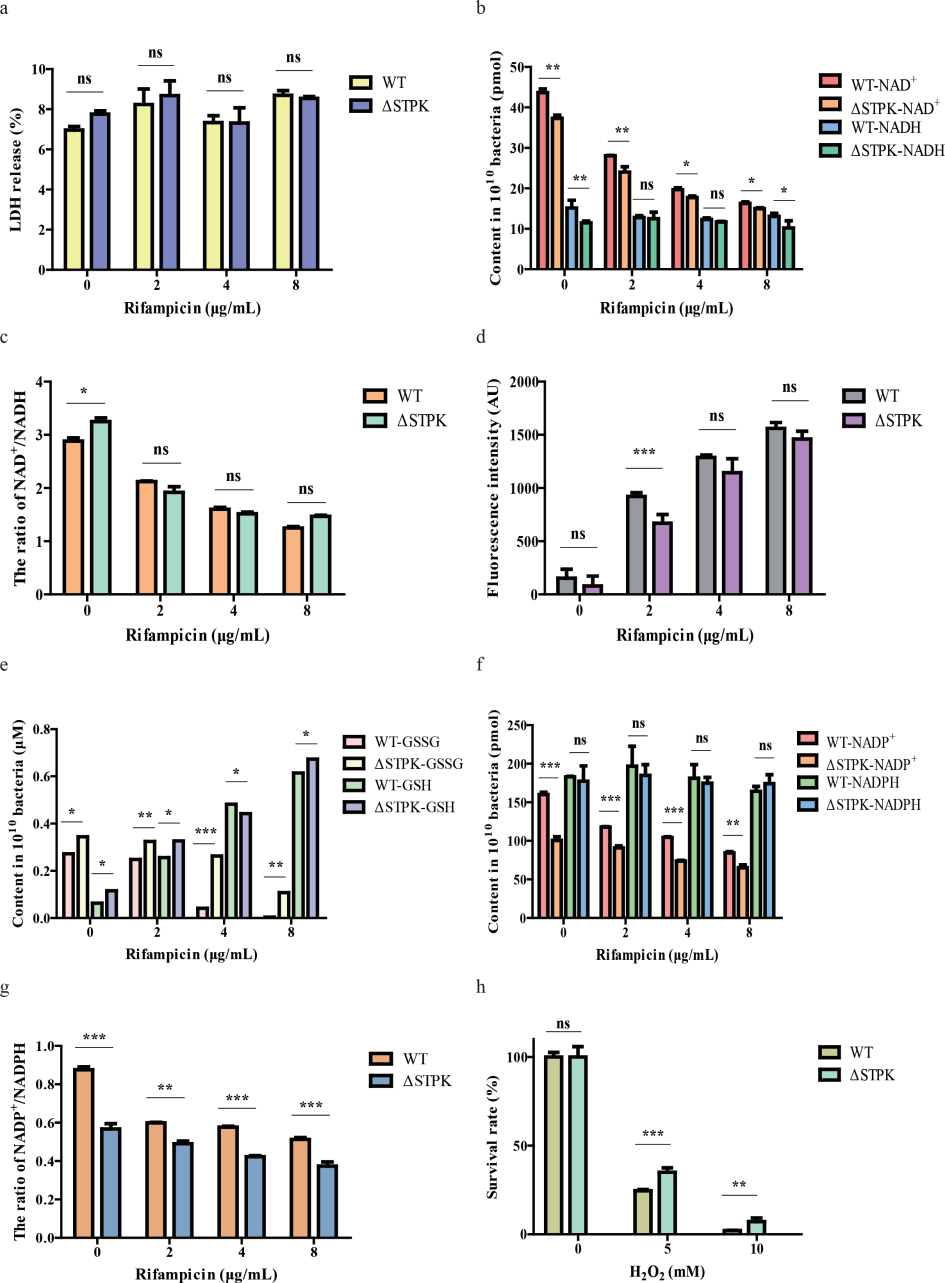
Biochemical parameters assay of WT and ΔSTPK strains under different rifampicin concentrations. (a) The LDH release in the culture supernatant of WT and ΔSTPK strains induced by different concentrations of rifampicin. (b) The NAD^+^ and NADH levels, (c) the ratio of NAD^+^/NADH, (d) the ROS level, (e) the total GSH and GSH content, (f) the NADP^+^ and NADPH content, (g) the ratio of NADP^+^/NADPH in WT and ΔSTPK strains. (h) The survival rates of WT and ΔSTPK strains under 0, 5, and 10 mM H_2_O_2_ conditions. Significance levels: ns, *P* > 0.05; *P < 0.05; **P < 0.01; ***P < 0.001.

To elucidate the mechanism of rifampicin resistance involved in STPK, this study measured the biochemical parameters between WT and ΔSTPK strains. It was shown that there was no significant difference in LDH release at different concentrations of rifampicin (Fig. 3a). The NAD^+^/NADH ratio in the ΔSTPK strains was altered compared to the WT strain, but the differences between WT and ΔSTPK strains were not significant at different rifampicin concentrations (Fig. 3b and c). These results showed that energy metabolism was decreased when STPK was deleted in *B. melitensis*. The ROS levels in both WT and ΔSTPK strains increased with higher rifampicin concentrations, but the ΔSTPK strain had lower ROS levels compared to the WT strain (Fig. 3d). Notably, total glutathione (GSH and GSSG) levels were higher in the STPK deletion strain compared to the wild type (Fig. 3e). The ΔSTPK strain exhibited higher NADPH levels than the WT strain, and significant differences in the NADP^+^/NADPH ratio were observed under different rifampicin concentrations, indicating enhanced ROS clearance ability in the deletion strain (Fig. 3f and g). Additionally, the ΔSTPK strain showed higher survival rates in oxidative environments compared to the WT strain (Fig. 3h), indicating enhanced antioxidant capacity in the deletion strain. All these findings indicate that the absence of the STPK could increase sulfur metabolism and GSH levels, and decrease the NADPH oxidase activity and NADP^+^/NADPH ratio, which promotes the antioxidant capacity of *B. melitensis*.

### Interaction between STPK and ribosomal protein RpsDin *B. melitensis*

**Fig 4:**
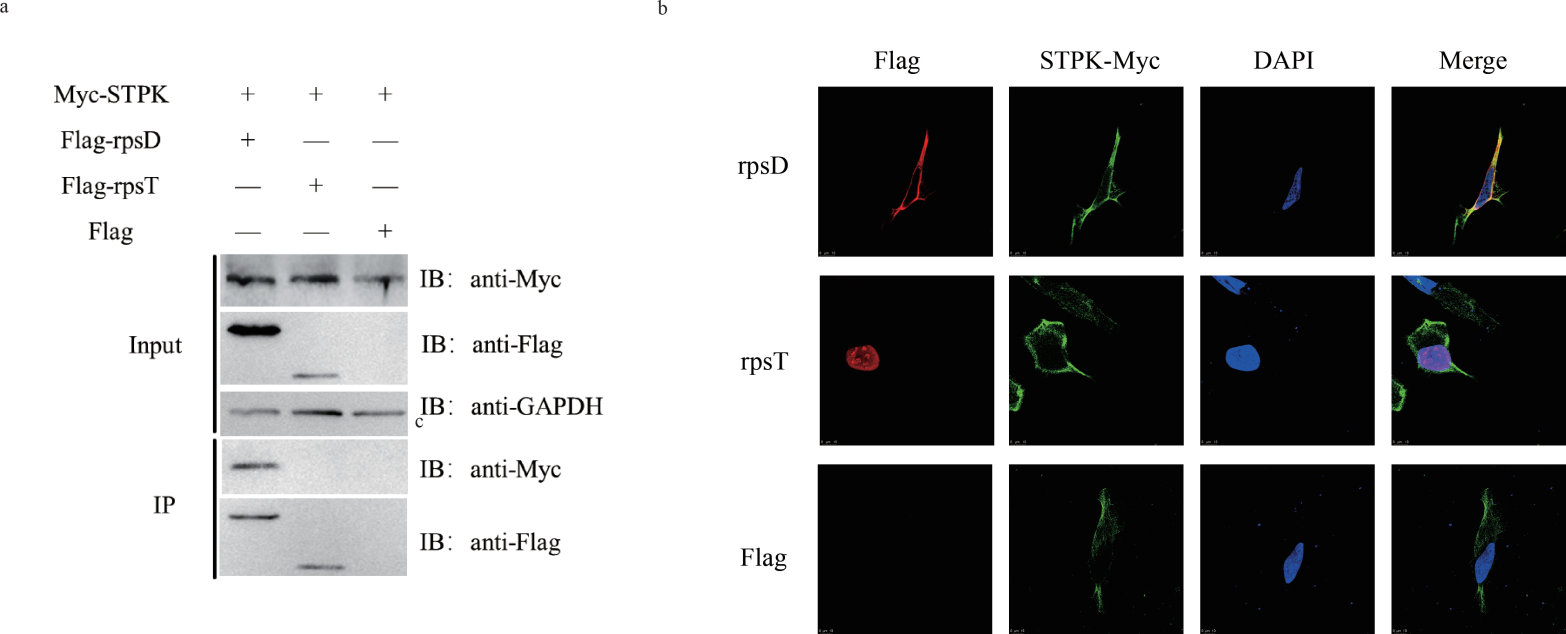
Interaction between STPK and ribosomal protein rpsD. (a) The results of Co-IP assay showing the interaction between STPK protein and ribosomal protein rpsD, with no interaction between STPK and ribosomal protein rpsT. (b)The results of laser confocal microscopy images showing co-localization of STPK protein and rpsD protein in the cytoplasm, with no co-localization between STPK and rpsT proteins.

To further investigate the mechanism of STPK-affected antioxidant capacity of *B. melitensis*, this study screened and identified the interacting protein of STPK through pull-down assays, LC-MS/MS, Co-IP, and confocal laser scanning microscopy analysis. After optimization of expression conditions, the STPK protein was expressed *in vitro* (Fig. S1c). Pull-down and LC-MS/MS experiments identified several interacting proteins. KEGG functional classification was used to select relevant proteins for Co-IP assay based on transcriptome results. Co-IP results showed that RpsD interacts with STPK protein (Fig. 4a), while the other expressed ribosomal proteins did not interact with STPK. Confocal laser microscopy revealed that RpsD co-localizes with the STPK protein in the cytoplasm of HeLa cells (Fig. 4b). All these indicate that STPK alters protein translation and riboswitch expression through interacting with the ribosomal protein RpsD.

### Molecular mechanism of STPK-mediated rifampicin resistance

**Fig 5:**
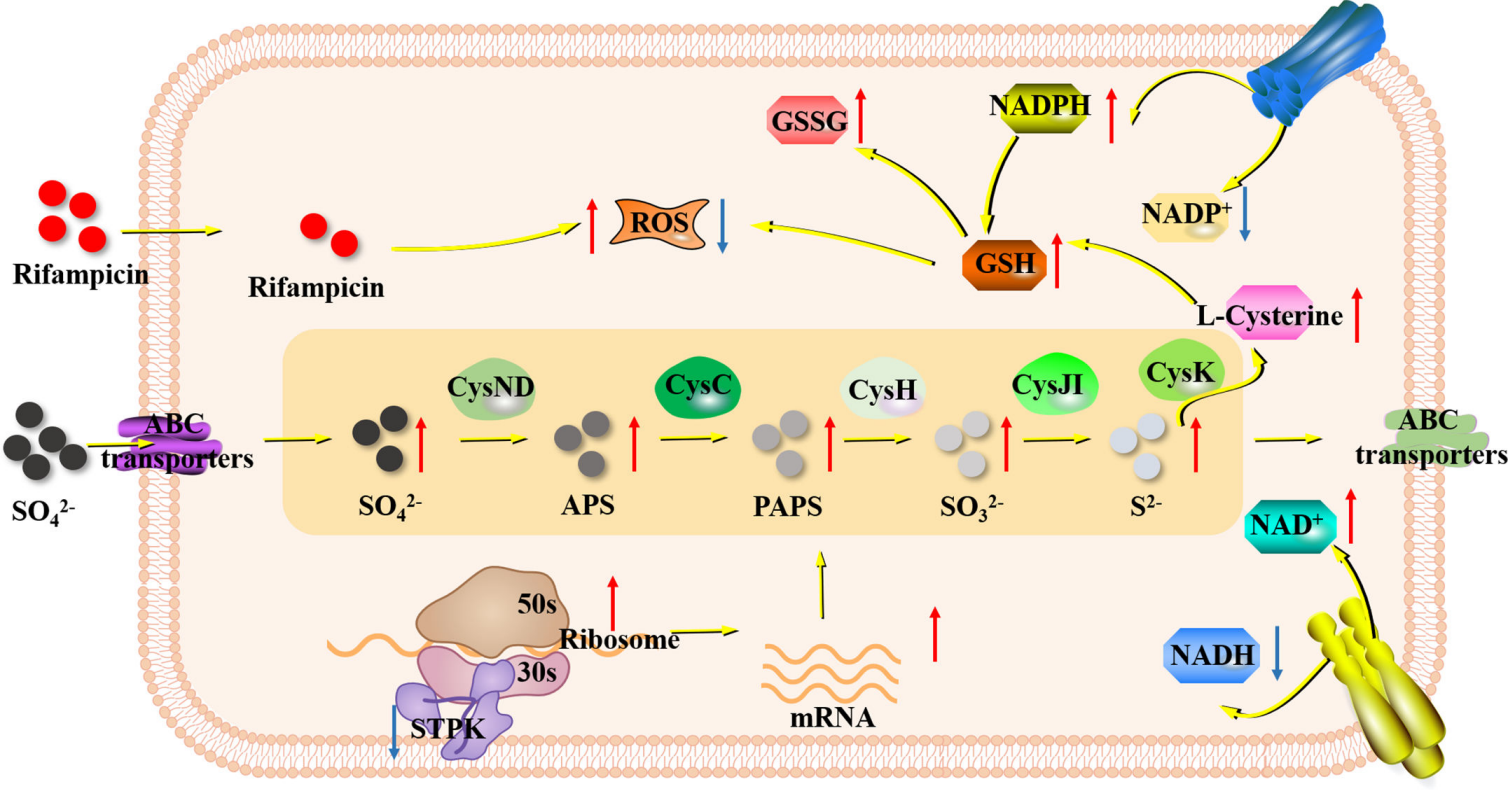
Molecular mechanism of STPK-mediated rifampicin resistance in *B. melitensis*. Yellow arrows are related pathways, red arrows are products with increased expression, and blue arrows are products with decreased expression. The orange box shows the sulfur metabolism of *B. melitensis*. After rifampicin interacts with *Brucella* strains, it triggers *Brucella* to produce a large amount of ROS. When the STPK gene was deleted (i.e., the expression of the STPK gene decreased), the expression of *rpsD* gene and ribosome-related gene increased. The energy metabolism was blocked, and the levels of NADH decreased. At the same time, ABC transport system transported more sulfate. The expression of sulfur metabolism-related genes increased, producing more GSH to resist the damage caused by ROS. During this process, the levels of NADPH also increased, which also helps in resisting ROS.

In summary, STPK gene mediates rifampicin resistance in *B. melitensis* through the following mechanisms (Fig. 5). (i) In protein interaction, the STPK gene interacts with ribosomal proteinRpsD, affecting protein translation and riboswitches expression. (ii) In gene deletion effects, deletion of the STPK gene leads to increased expression of ribosomal proteins, enhanced sulfur metabolism, elevated levels of total glutathione (GSSG and GSH), decreased NADPH oxidase activity, and a reduced NADP^+^/NADPH ratio. (iii) In enhanced antioxidant capacity, these changes enhance the antioxidant capacity of the STPK deletion strain, conferring resistance to damages caused by rifampicin-induced ROS, resulting in rifampicin resistance.

## DISCUSSION

This study found that the novel gene STPK is related to rifampicin resistance of *B. melitensis*. The STPK gene in *B. melitensis* encodes a serine/threonine protein kinase. Bioinformatics predictions indicate that this protein lacks a signal peptide (https://services.healthtech.dtu.dk/services/SignalP-5.0/) and transmembrane domains (https://services.healthtech.dtu.dk/services/TMHMM-2.0/), and is localized to the bacterial inner membrane (27). Although its function has not been previously studied in *Brucella* spp., STPK proteins with classical Hanks’ sequences are commonly found in prokaryotes (28). For instance, in *Streptomyces toyocaensis*, the STPK gene enhances glycopeptide antibiotic resistance by affecting the oxidative stress response (29). In *Deinococcus radiodurans*, the STPK gene has been shown to reduce DNA damage and regulate cell division (30). In *Mycobacterium tuberculosis*, the STPK gene was an important regulator responding to multiple environmental stresses (31). Additionally, *B. abortus* can activate host serine/threonine kinases during infection, inhibiting host NADPH oxidase activity to suppress ROS production and promoting intracellular survival (32). This study found that rifampicin induces ROS production in *Brucella* spp. The STPK deletion strain exhibited lower ROS levels compared to the wild-type strain, suggesting that STPK influences the oxidative stress response of *B. melitensis. In vitro* experiments confirmed this hypothesis, showing that the STPK deletion strain had a higher survival rate under oxidative conditions.

Transcriptome and qPCR analyses revealed that STPK deletion upregulated genes related to sulfur metabolism, resulting in increased total glutathione (GSSG and GSH). In bacteria, SO_4_^2−^ can be reduced to synthesize cysteine (33), which is further converted to glutathione (34). Both eukaryotic and prokaryotic cells utilize GSH in oxidative stress responses (35), explaining the enhanced antioxidant capacity of the STPK deletion strain due to higher GSH levels. STPK deletion also impaired the energy metabolism of *B. melitensis*, as evidenced by downregulated genes involved in valine, leucine, and isoleucine degradation, beta-alanine metabolism, and propanoate metabolism. However, no significant difference in energy metabolism was observed between the WT and STPK deletion strains under different rifampicin concentrations. Therefore, STPK affects the oxidative stress response of *B. melitensis* by influencing sulfur metabolism.

This study revealed that STPK interacts with the RpsD protein. The *rpsD* gene encodes ribosomal protein S4 of the 30S ribosomal subunit, which acts as a translational repressor (36, 37), and is a potential target for antibiotic development (38). This study found that STPK deletion increased *rpsD* gene expression, suggesting that STPK negatively regulates *rpsD*. Although increased expression of *rpsD* and sulfur metabolism-related genes was observed in the STPK deletion strain, no direct interaction between *rpsD* gene and sulfur metabolism genes was identified. Since the *rpsD* gene is involved in mRNA translation, riboswitches located in the 5′-UTR of mRNA can control gene expression (39) and regulate various cellular processes, including sulfur metabolism (40). This study hypothesizes that STPK interacts with RpsD protein, influencing the expression of ribosomal protein S4 and subsequently affecting mRNA and riboswitch expression, thereby regulating sulfur metabolism.

The results of this study hold significant public health implications for the prevention and control of brucellosis. Current studies indicate that rifampicin-resistant strains without *rpoB* gene mutations have been isolated worldwide (13, 41, 42). The STPK gene identified in this study can serve as a candidate target gene. Methods can be established based on this gene to study rifampin resistance in *B. melitensis*. If there is a deletion or mutation of the STPK gene in the isolated strain and the strain is resistant to rifampicin, detecting this gene can help determine whether patients can be effectively treated with rifampicin for brucellosis.

The transposon mutant library is a valuable tool for identifying new resistance genes. Our lab has previously used this library to identify *Brucella* genes conferring resistance to antibiotics such as trimethoprim-sulfamethoxazole and streptomycin. This study also identified multiple genes related to rifampicin resistance (unpublished data) using this library.

### Conclusion

In summary, this is the first demonstration that the deletion of STPK in *B. melitensis* mediates rifampicin resistance by enhancing antioxidant capacity. Moreover, the identified STPK can interact with the ribosomal protein RpsD, which might affect protein translation and riboswitch expression. However, whether STPK or its pathway can serve as an efficient target for new drug development against rifampicin-resistant *Brucella* spp. need to be further investigated.

## Data Availability

The transcriptome data of wild-type and STPK gene deletion strains have been deposited at the Sequence Read Archive under BioProject ID PRJNA1181107. Other data sets used and/or analyzed during the current study are available from the corresponding author upon reasonable request.

## SUPPLEMENTAL MATERIAL

Figure S1 msystems.01109-24-s0001.docx Flow chart of screening process and results of PCR and WB analysis.

Supplemental tables msystems.01109-24-s0002.xlsx Tables S1 to S4.

ASM does not own the copyrights to Supplemental Material that may be linked to, or accessed through, an article. The authors have granted ASM a non-exclusive, world-wide license to publish the Supplemental Material files. Please contact the corresponding author directly for reuse.
