# Supplementary material for: Serine/threonine protein kinase mediates rifampicin resistance in Brucella melitensis through interacting with ribosomal protein RpsD and affecting antioxidant capacity
Source: mSystems. 2024 Dec 5;10(1):e01109-24. doi: 10.1128/msystems.01109-24 (PMC11748488; doi:10.1128/msystems.01109-24)
Supplement: Figure S1 — Flow chart of screening process and results of PCR and WB analysis. [file msystems.01109-24-s0001.docx]

**
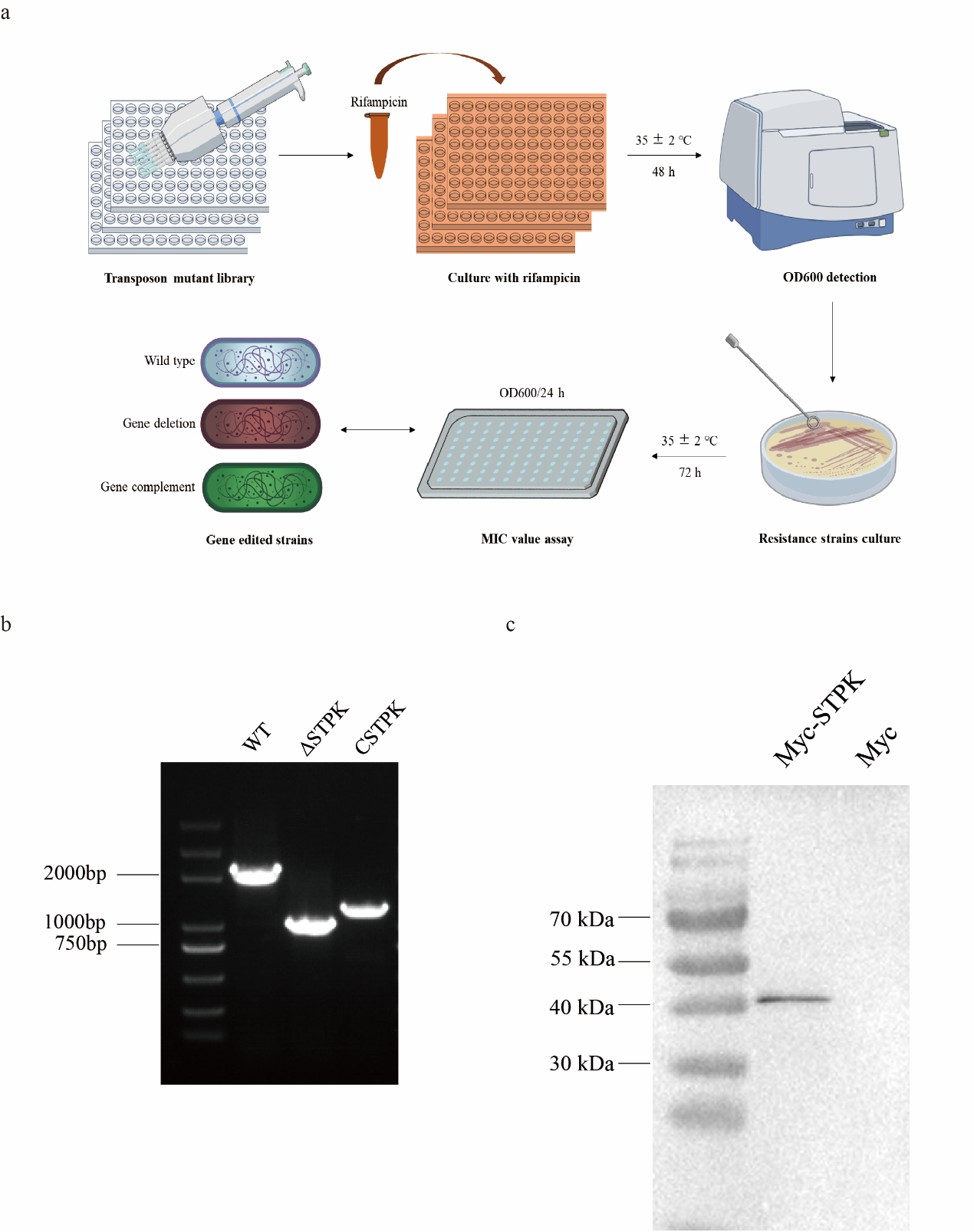
**

Figure S1 Supplementary figures of this study. (a) is the flowchart of the process for screening rifampicin resistance gene in this section of the experiment. (b) is PCR analysis of gene deletion and complementation. ΔSTPK strain was detected using primers for upstream and downstream fragments, and CSTPK strain was detected using plasmid-determined primers. (c) is the WB analysis of eukaryotic expression of STPK protein *in vitro*.
